# Quantitative MRI evaluation of gastric motility in patients with Parkinson’s disease: Correlation of dyspeptic symptoms with volumetry and motility indices

**DOI:** 10.1371/journal.pone.0216396

**Published:** 2019-05-03

**Authors:** Jungheum Cho, Yoon Jin Lee, Young Hoon Kim, Cheol Min Shin, Jong-Min Kim, Won Chang, Ji Hoon Park

**Affiliations:** 1 Department of Radiology, Seoul National University Bundang Hospital, Seongnam-si, Gyeonggi-do, South Korea; 2 Seoul National University College of Medicine, Seoul, South Korea; 3 Department of Gastroenterology, Seoul National University Bundang Hospital, Seongnam-si, Gyeonggi-do, South Korea; 4 Department of Neurology, Seoul National University Bundang Hospital, Seongnam-si, Gyeonggi-do, South Korea; University of Malaya Faculty of Medicine, MALAYSIA

## Abstract

**Objective:**

To investigate the correlation between dyspeptic symptoms and gastric motility parameters measured by magnetic resonance imaging (MRI) using volumetry and motility indices in patients with Parkinson’s disease (PD).

**Materials and methods:**

In this IRB-approved study, MRI datasets obtained from August 2014 to May 2016 in 38 PD patients were retrospectively analyzed. Patients underwent a 120-minute-long MRI study using a liquid test meal and 8 sets of scans. Gastric content volume (GCV) and total volume (TGV), gastric half emptying time (T_1/2_), motility index (GMI), accommodation (GA), and emptying (GE) values were acquired. These measurements were compared between patients according to the presence of gastric symptoms: early satiety (n = 25), epigastric pain (n = 13), and dyspepsia (n = 28).

**Results:**

Patients with early satiety showed significantly decreased GE of GCV and TGV (*p* < 0.001 and *p* = 0.017). Dyspeptic patients had significantly decreased GE of GCV and GMI (*p* = 0.001 and *p* = 0.029). GE of GCV at 90 and 120 minutes were significantly lower in patients with early satiety (*p* = 0.001 and *p* = 0.002). GE of GCV and GMI at 90 minutes were significantly decreased in patients with dyspepsia (*p* = 0.004 and *p* = 0.002). T_1/2_ of GCV was prolonged in patients with early satiety, epigastric pain, and dyspepsia (*p* = 0.004, *p* = 0.041, and *p* = 0.023). T_1/2_ of TGV also delayed in patients with early satiety (*p* = 0.023). GMI at 90 minutes was significantly correlated with dyspepsia on multivariable analysis (*p* = 0.028).

**Conclusions:**

Gastric motility can be quantitatively assessed by MRI, showing decreased GMI, delayed GE, and prolonged T_1/2_ in PD patients with early satiety or dyspepsia.

## Introduction

Parkinson’s disease (PD) is the second most prevalent degenerative disease in people above 65 years, and affects 1% of the population above 60 years [1]. Gastrointestinal dysfunction is frequent in PD, involving approximately 30% of patients [2]. Symptoms include dysphagia, nausea, bloating, delayed gastric emptying, and constipation, which are thought to be due to decreased gastrointestinal motility. Of these, gastroparesis is a significant problem in PD patients [3], and has a detrimental effect on regulation of the level of levodopa, which is absorbed only in the small bowel [4, 5]. However, there are few studies on the quantitative assessment of impaired gastric motility in PD patients [6, 7].

Unlike structural diseases manifested as fixed stenosis or obstruction which can be evaluated by computed tomography, functional impairment of gastric motility is a challenging disease to assess with conventional imaging studies taken at a single time point. Gastric scintigraphy, which is the gold standard for evaluating gastric emptying, has drawbacks such as radiation exposure and limited temporal and spatial resolution [8]. Other assessment techniques such as gastric barostat [9], ultrasound imaging [10], and single photon emission computed tomography [11] which have been used for measuring gastric accommodation, have shortcomings of invasiveness [12], subjectiveness [13], and radiation exposure [14], respectively. On the other hand, according to previous studies [8, 15], magnetic resonance imaging (MRI) which is non-invasive and has no radiation exposure can allow simultaneous morphologic and functional evaluation of the stomach.

Although there were some reports on MRI assessment of impaired gastric function in PD patients [6] and in diabetic patients [16], there is no study comparing gastric functional MRI measurements with clinical aspects in PD patients. Therefore, this study aims to investigate the correlation between dyspeptic symptoms and gastric motility parameters measured by MRI using volumetry and motility indices in PD patients.

## Materials and methods

This study was approved by the Institutional Review Board of Seoul National University Bundang Hospital (B-1712-436-103) and requirement of informed consent was waived (The data were analyzed anonymously). This study was conducted according to the Declaration of Helsinki.

### Study population

**Fig 1:**
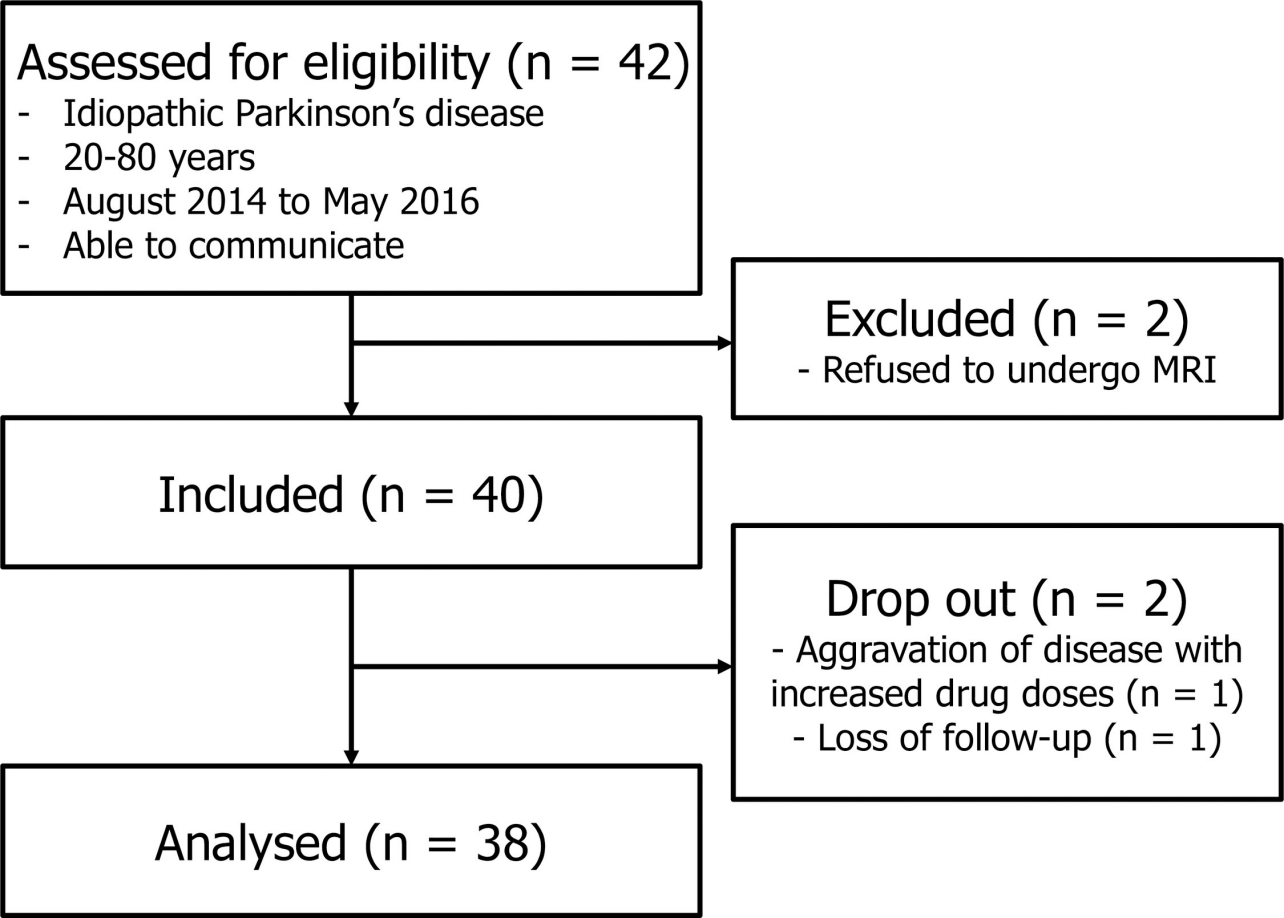
Flow diagram of enrolled patients. The analyzed patients were divided into three groups according to the presence of gastric symptoms: early satiety (n = 25), epigastric pain (n = 13), and dyspepsia (n = 28).

Patient data were collected prospectively for a randomized controlled trial testing the non-inferiority of a novel prokinetic drug [17], which included patients diagnosed as idiopathic PD by UK Parkinson’s disease Society Brain Bank criteria [18] with age of 20–80 years, from August 2014 to May 2016. This study is aimed to retrospectively compare various measurements on baseline MRI obtained before taking the drug, according to the presence of dyspeptic symptoms. Of the 42 patients who were assessed for eligibility, two patients were excluded for refusing to undergo MRI (n = 2). During the study, two patients dropped out due to loss of follow-up (n = 1) and aggravation of disease requiring increased drug doses for PD (n = 1). Finally, 38 patients were included for this retrospective analysis (Fig 1). Basic clinical information including body weight index (BMI), duration of PD, levodopa equivalent daily dose (LEDD), and medication duration were extracted from the prospectively collected database. Enrolled participants also answered the unified Parkinson's disease rating scale (UPDRS) questionnaire [19] and filled a gastrointestinal symptom diary [20].

For analysis, patients were divided into groups according to the presence of dyspeptic symptoms; early satiety, epigastric pain, and dyspepsia. Dyspepsia was defined as present if either early satiety or epigastric pain was present.

### MRI protocol

After at least 6 hours of fasting, participants underwent MRI of the stomach in supine position, before and after drinking a 400-mL fluid meal (Nucare, 400 kcal, carbohydrate: protein: fat = 57:15:27; Daesang Wellife, Seoul, Korea). All images were obtained between 08:00 and 13:00 hours.

**Fig 2:**
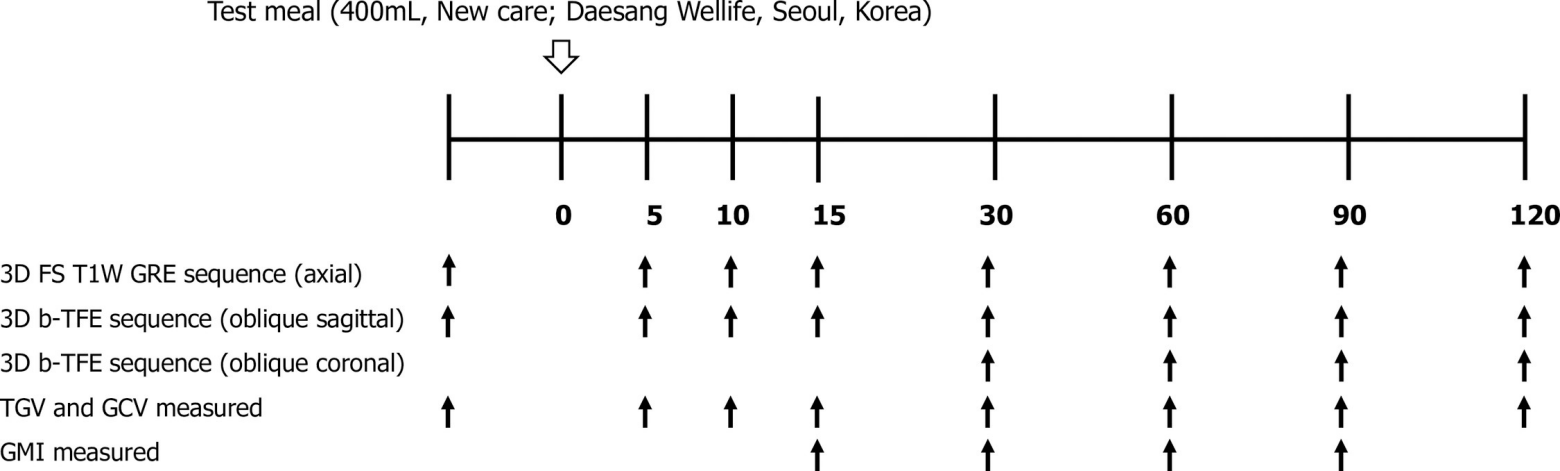
Timetable for obtaining MR images. MR images with axial 3D fat-suppressed T1-weighted gradient echo (GRE) sequence and oblique sagittal 3D balanced-turbo field echo (b-TFE) sequence were obtained before and after meal until 120 minutes. Oblique coronal 3D b-TFE sequence was also used from 30 minutes after meal. TGV, total gastric volume, GCV, gastric content volume, GMI, gastric motility index.

Patients were scanned with 3.0-T Ingenia or 1.5-T Intera MRI units (Philips Healthcare, Best, the Netherlands) with body coil (16-channel for Ingenia and 8-channel for Intera). Axial 3D fat-suppressed T1-weighted gradient echo sequence (mDixon for Ingenia and THRIVE for Intera) covering the stomach was used to measure the gastric volume before and after meal at 5, 10, 15, 30, 60, 90, and 120 minutes (Fig 2). For evaluation of proximal gastric motility, oblique sagittal images parallel to the long axis of gastric body were obtained before and after meal at 5, 10, 15, 30, 60, 90, and 120 minutes by using 3D balanced-turbo field echo (b-TFE) sequence. Distal stomach images were obtained at 30, 60, 90, and 120 minutes after meal by using the same b-TFE sequence with oblique coronal plane parallel to the long axis of gastric antrum. Further details of MR imaging parameters are listed in Table 1. We organized the MRI protocol according to previous studies and modified it according to our study purpose and institutional setting [6, 21].

**Table 1:** MR imaging parameters.

Sequence	3D FS T1-weighted GRE		3D b-TFE			
Scan plane	Axial		Oblique sagittal		Oblique coronal	
**Tesla**	3 T	1.5 T	3 T	1.5 T	3 T	1.5 T
**TR (ms)**	4.2	4.05	2.16	3.40	2.16	3.36
**TE (ms)**	1.45	1.96	1.08	1.70	1.08	1.68
**FOV (mm)**	320 × 320	315 × 350	400 × 400	272 × 340	400 × 400	360 × 360
**Matrix**	280 × 212	160 × 112	200 × 200	224 × 224	200 × 200	224 × 179
**ST (mm)**	5	5	10	10	10	10
**SI (mm)**	2.5	2.5	10	10	10	10
**Sense factor**	2	2	2	2	2	2
**Acquisition time**	10 seconds within one breath hold		3 minutes with free breathing, 1 second per image (30 images of 5 second intervals at 5 consecutive planes)			

FS, fat-suppressed, GRE, gradient echo, b-TFE, balanced-turbo field echo, TR, repetition time, TE, echo time, FOV, field-of-view, ST, slice thickness, SI, slice interval

### Image analysis

**Fig 3:**
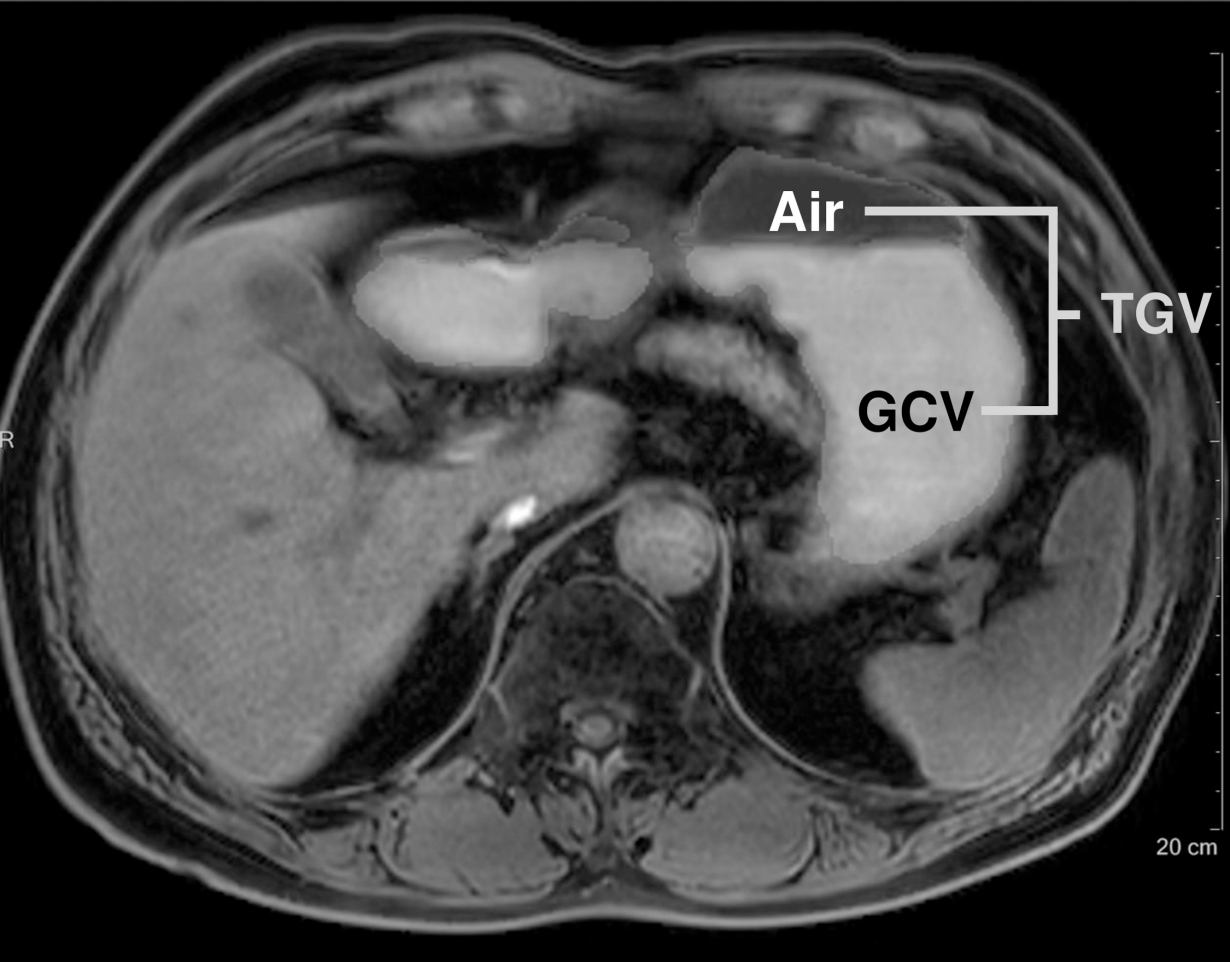
Gastric volume measurement of a 68-year-old man with Parkinson’s disease. In axial T1-weighted image, regions of interest (ROI) of gastric contents and air were drawn by a radiologist with volume analysis software. The volume of ROI was calculated automatically. TGV, total gastric volume, GCV, gastric content volume.

Gastric content volume (GCV) and total gastric volume (TGV) were measured by semi-automatic method using volume analysis software (Multi-Modality Tumor Tracking, Intellispace Portal version 5.0.2; Philips Healthcare, Best, the Netherlands) by one radiologist (Y.J.L. with 11 years of experience in body imaging). Regions of interest (ROIs) were drawn roughly along the margin of gastric contents and intraluminal air, respectively, on axial fat-suppressed T1-weighted images and the margin was carefully modified. Then, the volume of ROIs was calculated automatically by the software (Fig 3). TGV was defined as sum of gastric contents and air volume.

Gastric half emptying time (T_1/2_) of GCV and TGV were derived respectively from the power exponential model which was described by Elashoff et al [22].

**Fig 4:**
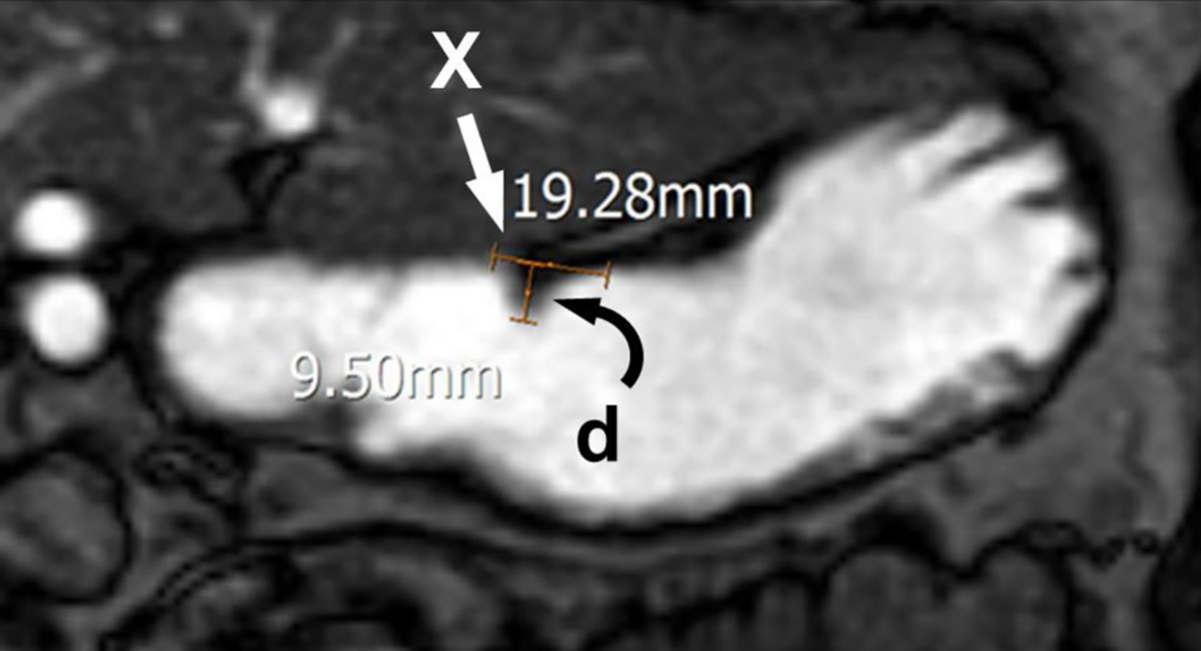
Measurements for calculating gastric motility index of a 68-year-old man with Parkinson’s disease. Wavelength (X, straight arrow) and amplitude (d, curved arrow) of gastric peristaltic waves were measured in the 3D balanced-turbo field echo MR image. Number of waves (not shown in this image) was also obtained from the same sequence. Gastric motility index was calculated based on these measurements.

Gastric motility indices (GMIs) at 15, 30, 60, and 90 minutes after meal were calculated from measurements in b-TFE images by one radiologist (J.C. with 3 years of experience in body imaging). The gastric wave was measured at the location where sinusoid wave was most prominently visible. Usually, it was measured at the proximal stomach in the 15-minute-delayed scan, and at the mid to distal stomach afterwards. Wavelengths, number of waves within serially obtained 30 images, and amplitudes of gastric peristaltic waves were measured on the b-TFE images (Fig 4). Wavelength was defined as the distance between the two end points of the inward deflection of the most prominent sinusoid wave. GMI was calculated for each participant using the following equation, which was modified from previously reported formula [6]:

$$\mathrm{GMI}\;[{mm}^{2}/s]=\frac{\mathrm{Wavelength}\times \mathrm{Number}\;\mathrm{of}\;\mathrm{waves}}{\mathrm{Total}\;\mathrm{scan}\;\mathrm{time}}\times \mathrm{Amplitude}$$

Gastric accommodation (GA) and gastric emptying (GE) at x minutes after ingestion of test meal (x = 5, 10, 15, 30, 60, 90, and 120 minutes) were defined as following formulas:

$$\mathrm{GA}=\frac{\mathrm{Gastric}\;\mathrm{volume}\;\mathrm{at}\;5\;\mathrm{minutes}‑\mathrm{Gastric}\;\mathrm{volume}\;\mathrm{before}\;\mathrm{ingestion}}{\mathrm{Gastric}\;\mathrm{volume}\;\mathrm{before}\;\mathrm{ingestion}}$$

$$\mathrm{GE}\;(\%)=100‑\frac{\mathrm{Gastric}\;\mathrm{volume}\;\mathrm{at}\;x\;\mathrm{minutes}‑\mathrm{Gastric}\;\mathrm{volume}\;\mathrm{before}\;\mathrm{ingestion}}{\mathrm{Gastric}\;\mathrm{volume}\;\mathrm{at}\;5\;\mathrm{minutes}‑\mathrm{Gastric}\;\mathrm{volume}\;\mathrm{before}\;\mathrm{ingestion}}\times 100$$

GA and GE of TGV and GCV were calculated respectively. The radiologists were blinded to the presence of dyspeptic symptoms while analyzing the images.

### Statistical analysis

Statistical analysis was performed by using SPSS version 19.0 software (IBM, Armonk, NY) and R version 3.3.2 software (R Foundation for Statistical Computing, Vienna, Austria). *P* < 0.05 was considered as statistically significant. Clinical features which might affect gastric parameters such as age, BMI, duration of PD, LEDD, and medication duration were analyzed using independent t-test between PD patients with and without dyspeptic symptoms. GE of GCV and TGV, and GMIs were compared between PD patients with and without dyspeptic symptoms, using repeated measures ANOVA, and *post hoc* analysis was done using independent t-test with Bonferroni correction at each timepoint. GA and T_1/2_ were also compared using Mann-Whitney tests. Generalized estimating equation was applied for multivariable analysis to determine significant independent clinical or imaging features that might be correlated with the presence of dyspeptic symptoms in PD patients. Clinical or imaging features which showed statistical significance in the aforementioned univariable analyses were used as independent variables for the generalized estimating equation.

## Results

All participants ingested the fluid meal and underwent MRI without complication. Table 2 shows the characteristics of included PD patients. Twenty-eight patients experienced dyspeptic symptoms related to meal; early satiety in 15 patients, epigastric pain in 3 patients, and both in 10 patients. The remaining 10 patients did not complain of any dyspeptic symptoms. There was no statistically significant difference of age, BMI, duration of PD, LEDD, and medication duration between PD patients with and without dyspeptic symptoms in each group (S1 Table).

**Table 2:** Patient demographics.

Characteristic		Value
Age (years)		69 (64–72)
	Male	70 (66–73)
	Female	68 (58–72)
Gender		
	Male (%)	20 (53)
	Female (%)	18 (47)
BMI (mean ± SD, kg/m2)		23.1 ± 3.0
UPDRS (mean ± SD)		22.3 ± 9.1
Disease duration (mean ± SD, months)		62.5 ± 45.6
LEDD (mean ± SD, mg/day)		635.5 ± 404.6
Duration with levodopa (mean ± SD, months)		49.8 ± 44.9
Patients with dyspeptic symptom (%)		28 (74)
	Early satiety (%)	25 (66)
	Epigastric pain (%)	13 (34)
MRI scanner		
	1.5-T (%)	7 (18)
	3.0-T (%)	31 (82)

Age is presented as median value with interquartile range in parentheses. BMI, body mass index, UPDRS, unified Parkinson's disease rating scale, LEDD, levodopa equivalent daily dose, SD, standard deviation

The range of measured GCV and TGV were 0.5–551.0 mL and 6.6–864.4 mL, respectively. The coefficients of variation, obtained from the ratio of gastric air volume to GCV, were between 0.6 and 1.3.

The mean T_1/2_ of GCV and TGV of 38 patients were 113.82 minutes (range 60.85–166.79) and 98.98 minutes (range 53.66–144.30), respectively.

**Fig 5:**
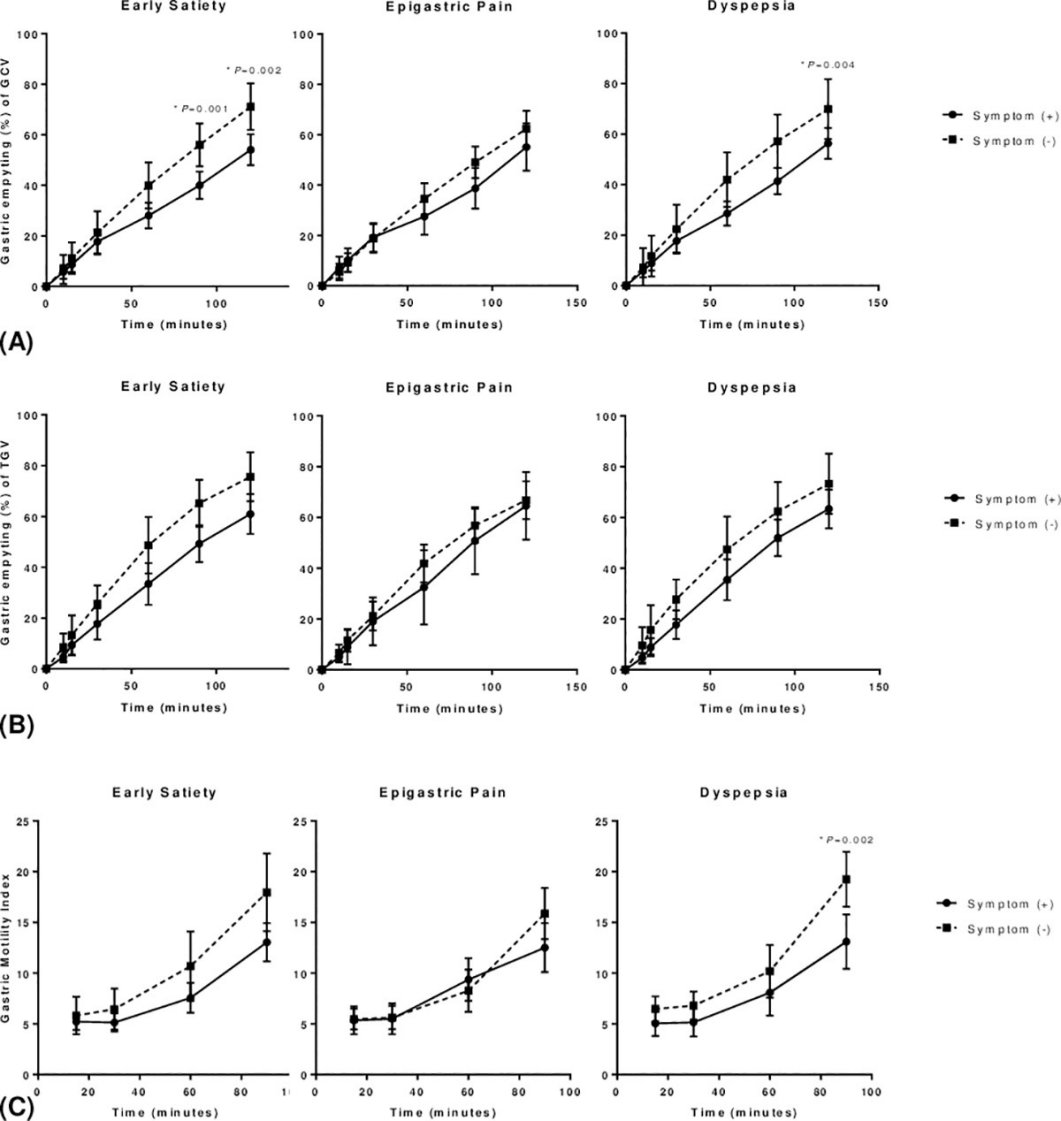
Line graphs of gastric emptying and gastric motility index in patients with each symptom. **A.** Gastric emptying of gastric content volume **B**. Gastric emptying of total gastric volume **C**. Gastric motility index, GCV, gastric content volume, TGV, total gastric volume, repeated measures ANOVA was used for comparison between patients with and without dyspeptic symptoms and *post hoc* analysis was done using independent t-test with Bonferroni correction.

Mean GE of GCV and TGV, and GMIs at each time points were compared between patients with and without the dyspeptic symptoms (Fig 5, Table 3, S2 and S3 Tables). Patients with early satiety showed significantly decreased GE of GCV and TGV (*p* < 0.001 and *p* = 0.017, respectively) compared with asymptomatic patients. For epigastric pain, there was a significant difference in GE of GCV between the two groups over time (*p* = 0.029). On the other hand, there was no statistically significant difference in GE of TGV and GMI between the patients with and without epigastric pain. Patients who had dyspepsia also showed significant decrease in the GE of GCV and GMI (*p* = 0.001 and *p* = 0.029, respectively). Independent t-test with Bonferroni correction showed significant difference of GE of GCV at 90 and 120 minutes (*p* = 0.001 and *p* = 0.002) between the patients with and without early satiety. GE of GCV and GMI at 90 minutes (*p* = 0.004 and *p* = 0.002, respectively) were significantly decreased in patients with dyspepsia.

**Table 3:** Comparison of gastric motility index in patients with or without dyspepsia.

	Dyspepsia (+)	Dyspepsia (-)	*p*-value
**15 minutes**	5.0 ± 2.1	6.8 ± 3.3	0.112
**30 minutes**	5.1 ± 2.3	6.8 ± 3.3	0.095
**60 minutes**	8.1 ± 3.8	10.2 ± 6.3	0.216
**90 minutes**	13.1 ± 4.4	19.3 ± 6.6	0.002*
**RM-ANOVA**^**†**^			0.029*

Except for P-value, data are presented as mean ± standard deviation.

*p < 0.05

† Results of repeated measures ANOVA

The comparison results of GA and T_1/2_ of GCV and TGV are presented in Table 4. GA did not show statistical difference between patients with and without dyspeptic symptoms. On the other hand, T_1/2_ of GCV was significantly prolonged in patients with early satiety, epigastric pain, or dyspepsia (*p* = 0.004, *p* = 0.041, and *p* = 0.023, respectively). T_1/2_ of TGV also prolonged in patients with early satiety (*p* = 0.023).

**Table 4:** Comparison of gastric accommodation and half emptying time.

		Gastric accommodation (GA)			Gastric half emptying time (T_1/2_)		
		Symptom (+)	Symptom (-)	*P-*value	Symptom (+)	Symptom (-)	*P-*value
Gastric content volume							
	**Early satiety**	101.67 ± 154.84	76.41 ± 85.54	0.879	145.98 ± 96.65	83.96 ± 32.18	0.004*
	**Epigastric pain**	57.00 ± 81.39	111.77 ± 153.23	0.246	165.98 ± 125.57	103.33 ± 44.14	0.041*
	**Dyspepsia**	94.60 ± 147.56	88.64 ± 94.59	0.909	139.22 ± 93.48	84.28 ± 35.52	0.023*
**Total gastric volume**							
	**Early satiety**	18.86 ± 18.77	22.09 ± 18.43	0.447	118.61 ± 78.69	63.70 ± 33.15	0.023*
	**Epigastric pain**	18.33 ± 18.88	20.81 ± 18.59	0.429	119.05 ± 69.72	97.63 ± 50.80	0.259
	**Dyspepsia**	19.81 ± 20.56	20.41 ± 11.57	0.257	139.22 ± 93.48	113.26 ± 62.31	0.142

Except for P-value, data are presented as mean ± standard deviation.

*p < 0.05

Multivariable analysis showed one statistically significant MR imaging feature, GMI at 90 minutes (*p* = 0.028), in patients with and without dyspepsia (S4 Table).

## Discussion

In this study, we performed a quantitative MRI analysis using volumetry and motility indices and evaluated the correlation of dyspeptic symptoms and various MRI measurements in PD patients.

Gastric volume measurement by using MRI has been demonstrated to be reliable compared with other methods, such as barostat, gastric scintigraphy, or ^13^C-breath test [15, 23]. The non-invasive nature of MRI can provide a more tolerable method in GE and GA assessment, and is more capable of evaluating physiological characteristics encompassing both secretion and air in the stomach [24]. Moreover, gastric motility can be estimated visually and quantitatively by MRI at the same time [6, 25].

In our study, T_1/2_, GE, GA, and GMI were compared between patients with and without dyspeptic symptoms including early satiety, epigastric pain, and dyspepsia. The T_1/2_ of GCV and TGV in PD patients were prolonged compared with those of healthy volunteers in previous studies using MRI [21] and gastroscintigraphy [26]. This corresponds with several previous studies showing impaired gastric emptying in PD patients [6, 27]. Fauehauf et al. [28] demonstrated that there is a correlation between GCV estimated by MRI and fullness in healthy controls and patients with functional dyspepsia. These findings may suggest that retention of the gastric contents in the stomach could be one of the causes of dyspeptic symptoms such as fullness and epigastric discomfort. In accordance with previous studies, PD patients with early satiety showed delayed GE, prolonged T_1/2_, and decreased GMI compared to patients without the symptom in our study. To the best of our knowledge, this is the first study to correlate dyspeptic symptoms and gastric MRI measurements in PD patients.

On the other hand, there was no statistically significant difference of gastric measurements between PD patients with and without epigastric pain, except for the overall trend of decreased GE of GCV. This could be attributed to the pathophysiology of delayed gastric emptying in PD patients. Although it is not clearly understood, the neuronal and hormonal abnormalities in PD are due to alpha-synuclein deposition in central (dorsal motor nucleus of the vagus nerve) and enteric nervous system. In PD patients, the stomach is one of the primary sites of alpha-synuclein deposition [29]. Therefore, decreased motility of the stomach or gastroparesis, rather than gastric outlet obstruction or stenosis, may be accompanied by early satiety, rather than epigastric pain. This is in keeping with a previous study reporting higher prevalence of bloating and nausea than abdominal pain in PD patients [30].

There is scarce data on the optimal timing to notice a significant difference by gastric imaging to reflect gastric dysfunction. The difference of various MRI measurements between the groups was prominent after 90 minutes in this study. This is in line with a previous study which reported a gastric half emptying time of PD patients with delayed gastric emptying to be 89.5 minutes on liquid meal gastric scintigraphy [23]. We also observed a trend of increasing GMI until 90 minutes after ingestion. Although there is no such study measuring GMI timely by MRI, it is consistent with a previous study showing maximal gastric emptying rate at 119 minutes after ingestion of liquid meal containing [^13^C]glycine [31]. Based on our results and previous studies, it may be necessary to obtain images until at least 90 minutes after ingestion of test meal to observe a significant difference in future studies assessing impaired gastric function of PD patients.

In terms of MRI volumetry, GCV seems to be better correlated with dyspeptic symptoms than TGV in PD patients. This finding may be owing to the technical difficulty in measuring the air volume due to susceptibility artifacts on MR images. In addition, the relative amount of air ingested in each patient showed great variability compared to the gastric content in our study, with coefficient of variance between 0.6 and 1.3, which also may dilute the potentially significant differences between certain groups.

As GMI at 90 minutes was the only significant imaging feature for dyspepsia on multivariable analysis, future studies on imaging assessment of gastric motility should include similar motility indices to overcome the simplicity of volumetry alone and offer more comprehensive information. Although we have adopted a rather plain measurement of gastric peristaltic waves, more sophisticated techniques such as motility mapping or gastrointestinal tagging may be of value and provide promising results for evaluating dyspepsia [32].

When assessing bowel motility by imaging, it is crucial to control the clinical factors which might influence the motility. According to previous studies [26, 33], UPDRS is one of the confounding factors in evaluation of gastric emptying of PD patients, unlike age, sex, and BMI. However, in this study, there was no statistically significant difference of UPDRS among all of the compared groups. Although there is a possibility that levodopa medication in our patients had various effects on UPDRS and gastric motor function, the number of patients in our study might have been too small to have sufficient statistical power as it was originally designed to show the non-inferiority of a new prokinetic drug [17].

There are several limitations in this study. First, the sample size in this retrospective study was predetermined for a prospective clinical trial with a different study purpose. The numbers of patients in each symptom group might not be optimal to show a statistically significant difference by MRI volumetry or motility indices. A total of 36 patients were required for the prospective clinical trial from *a priori* power analysis with a non-inferiority limit of -10%, based on a previous study reporting the standard deviation of GE rate at 120 minutes to be 10% in healthy subjects [21]. Nonetheless, as the significant differences in GE of GCV in certain comparison groups exceed 10%, our results can still provide valuable information. Moreover, we were able to show a general trend of decreased GE or GMI in symptomatic patients, especially with early satiety. In addition, our study included more patients than previous reports on gastric motility [6, 15, 16, 21, 25]. A future prospective study with a larger number of patients may be warranted. Secondly, fluid meal was used for evaluating gastric volume. Although it is not a routine meal for patients, it is convenient for ingestion and enables better visualization with less susceptibility artifacts on MRI. Many studies showed that it is feasible to evaluate gastric function with MRI using a liquid meal [21, 23, 34–36] or a semi-solid meal such as pudding or scrambled egg with water [6, 25]. Gastric emptying measured by gastric scintigraphy is generally known to be delayed for solid meals compared to liquid meals [37]. Therefore, the usage of a solid test meal might have resulted in fewer numbers of significant MRI-derived parameters obtained within 120 minutes after ingestion. Accordingly, our results should be interpreted with attention. Thirdly, we were not able to compare the data between healthy volunteers and PD patients, which could have given important information to understand the pathophysiology of PD.

In conclusion, gastric motility can be quantitatively assessed by MRI, showing decreased GMI, delayed GE, and prolonged T_1/2_ in PD patients with early satiety or dyspepsia. Therefore, MRI may become a comprehensive tool providing objective parameters for the evaluation of impaired gastric function in dyspeptic patients.

## Supporting information

S1 Table Comparison of clinical features between patients with and without symptoms. (DOCX)

S2 Table Comparison of gastric emptying between patients with and without early satiety. (DOCX)

S3 Table Comparison of gastric emptying between patients with and without epigastric pain or dyspepsia. (DOCX)

S4 Table Results of generalized estimating equation. (DOCX)

## Abbreviations

PD: Parkinson’s Disease
GCV: Gastric Content Volume
TGV: Total Gastric Volume
T_1/2_: Gastric half emptying time
GMI: Gastric Motility Index
GA: Gastric Accommodation
GE: Gastric Emptying
