# Supplementary material for: Quantitative MRI evaluation of gastric motility in patients with Parkinson’s disease: Correlation of dyspeptic symptoms with volumetry and motility indices
Source: PLoS One. 2019 May 3;14(5):e0216396. doi: 10.1371/journal.pone.0216396 (PMC6499432; doi:10.1371/journal.pone.0216396)
Supplement: S1 Table — (DOCX) [file pone.0216396.s001.docx]

**S1 Table. Comparison of clinical features between patients with and without symptoms**

|  | Early satiety | | | Epigastric Pain | | | Dyspepsia | | |
| --- | --- | --- | --- | --- | --- | --- | --- | --- | --- |
|  | Symptom (+) | Symptom (-) | *P-*value | Symptom (+) | Symptom (-) | *P-*value | Symptom (+) | Symptom (-) | *P-*value |
| Age (mean ± SD, years) | 67 ± 7.2 | 69 ± 6.7 | 0.4 | 66 ± 6.3 | 68 ± 7.3 | 0.37 | 67 ± 6.9 | 69 ± 7.2 | 0.28 |
| BMI (mean ± SD, kg/m2) | 23.4 ± 3.04 | 22.7 ± 2.87 | 0.54 | 23.2 ± 3.81 | 23.1 ± 2.49 | 0.95 | 23.2 ± 3.15 | 22.9 ± 2.49 | 0.8 |
| UPDRS (mean ± SD) | 21.8 ± 10.1 | 23.3 ± 7.02 | 0.63 | 23.4 ± 11.7 | 21.5 ± 7.51 | 0.47 | 21.6 ± 10.1 | 24.2 ± 5.13 | 0.32 |
| Disease duration (mean ± SD, months) | 65.0 ± 51.0 | 48.2 ± 25.3 | 0.18 | 57.1 ± 36.3 | 60.4 ± 48.6 | 0.83 | 63.0 ± 49.1 | 48.9 ± 25.5 | 0.4 |
| LEDD (mean ± SD, mg/day) | 666 ± 469 | 577 ± 243 | 0.53 | 721 ± 573 | 591 ± 287 | 0.45 | 646 ± 457 | 605 ± 212 | 0.78 |
| Duration with levodopa (mean ± SD, months) | 54.0 ± 48.6 | 34.2 ± 28.0 | 0.12 | 44.7 ± 36.3 | 48.6 ± 47.2 | 0.8 | 51.6 ± 47.6 | 35.2 ± 26.7 | 0.31 |

Note—SD, standard deviation, BMI, body mass index, UPDRS, unified Parkinson's disease rating scale, LEDD, levodopa equivalent daily dose
