# Supplementary material for: Quantitative MRI evaluation of gastric motility in patients with Parkinson’s disease: Correlation of dyspeptic symptoms with volumetry and motility indices
Source: PLoS One. 2019 May 3;14(5):e0216396. doi: 10.1371/journal.pone.0216396 (PMC6499432; doi:10.1371/journal.pone.0216396)
Supplement: S2 Table — (DOCX) [file pone.0216396.s002.docx]

**S2 Table. Comparison of gastric emptying between patients with and without early satiety**

|  | Early Satiety | | | | | |
| --- | --- | --- | --- | --- | --- | --- |
|  | GE of GCV | | | GE of TGV | | |
|  | Symptom (+) | Symptom (-) | *P*-value | Symptom (+) | Symptom (-) | *P*-value |
| 10 minutes | 5.8 ± 6.6 | 6.7 ± 9.5 | 0.734 | 4.7 ± 5.1 | 8.6 ± 9.0 | 0.096 |
| 15 minutes | 8.7 ± 7.4 | 11.2 ± 10.4 | 0.400 | 9.3 ± 9.6 | 13.3 ± 12.9 | 0.291 |
| 30 minutes | 17.8 ± 11.5 | 21.2 ± 14.1 | 0.430 | 17.7 ± 14.9 | 25.6 ± 11.9 | 0.106 |
| 60 minutes | 28.0 ± 12.2 | 40.0 ± 15.0 | 0.012* | 33.4 ± 19.9 | 48.6 ± 18.5 | 0.028* |
| 90 minutes | 40.0 ± 13.2 | 56.0 ± 14.0 | 0.001*^†^ | 49.3 ± 17.6 | 65.2 ± 15.4 | 0.009* |
| 120 minutes | 54.0 ± 14.8 | 71.1 ± 15.2 | 0.002*^†^ | 61.0 ± 19.2 | 75.6 ± 15.9 | 0.024* |
| RM-ANOVA^‡^ |  |  | < 0.001* |  |  | 0.017* |

Note–Except for P-value, data are presented as mean ± standard deviation, GE, gastric emptying, GCV, gastric content volume, TGV, total gastric volume

*p < 0.05

†After Bonferroni correction, p < 0.008

‡Results of repeated measures ANOVA.
