# Supplementary material for: Quantitative MRI evaluation of gastric motility in patients with Parkinson’s disease: Correlation of dyspeptic symptoms with volumetry and motility indices
Source: PLoS One. 2019 May 3;14(5):e0216396. doi: 10.1371/journal.pone.0216396 (PMC6499432; doi:10.1371/journal.pone.0216396)
Supplement: S3 Table — (DOCX) [file pone.0216396.s003.docx]

**S3 Table. Comparison of gastric emptying between patients with and without epigastric pain or dyspepsia**

|  | Epigastric Pain | | | | Dyspepsia | | | |
| --- | --- | --- | --- | --- | --- | --- | --- | --- |
|  | GE of GCV | | | GE of TGV | GE of GCV | | | GE of TGV |
|  | Symptom (+) | Symptom (-) | *P*-value |  | Symptom (+) | Symptom (-) | *P*-value |  |
| 10 minutes | 7.3 ± 7.1 | 5.5 ± 7.9 | 0.504 |  | 5.7 ± 6.3 | 7.2 ± 10.9 | 0.604 |  |
| 15 minutes | 10.2 ± 7.7 | 9.2 ± 9.0 | 0.738 |  | 8.8 ± 7.3 | 11.7 ± 11.3 | 0.362 |  |
| 30 minutes | 19.3 ± 9.5 | 18.8 ± 13.9 | 0.905 |  | 17.8 ± 12.0 | 22.4 ± 13.5 | 0.317 |  |
| 60 minutes | 27.5 ± 12.0 | 34.5 ± 15.0 | 0.152 |  | 28.6 ± 12.4 | 41.9 ± 15.1 | 0.009* |  |
| 90 minutes | 38.7 ± 13.3 | 49.0 ± 15.4 | 0.048* |  | 41.3 ± 13.4 | 57.1 ± 14.8 | 0.004*^†^ |  |
| 120 minutes | 55.1 ± 15.6 | 62.4 ± 17.3 | 0.214 |  | 56.3 ± 15.8 | 69.9 ± 16.6 | 0.027* |  |
| RM-ANOVA^‡^ |  |  | 0.029* | 0.335 |  |  | 0.001* | 0.580 |

Note–Except for P-value, data are presented as mean ± standard deviation, GE, gastric emptying, GCV, gastric content volume, TGV, total gastric volume

*p < 0.05

†After Bonferroni correction, p < 0.008

‡Results of repeated measures ANOVA.
