# Supplementary material for: Quantitative MRI evaluation of gastric motility in patients with Parkinson’s disease: Correlation of dyspeptic symptoms with volumetry and motility indices
Source: PLoS One. 2019 May 3;14(5):e0216396. doi: 10.1371/journal.pone.0216396 (PMC6499432; doi:10.1371/journal.pone.0216396)
Supplement: S4 Table — (DOCX) [file pone.0216396.s004.docx]

**S4 Table. Results of generalized estimating equation**

|  | Early Satiety | Dyspepsia |
| --- | --- | --- |
|  | *p*-value | *p*-value |
| GE of GCV at 90 min | 0.892 | 0.338 |
| T_1/2_ of GCV | 0.099 | 0.180 |
| GMI at 90 min | - | 0.028* |

Note–GE, gastric emptying, T_1/2_, half emptying time, GCV, gastric content volume, GMI, gastric motility index

**p* < 0.05
